# Development of Haploid Plants by Shed-Microspore Culture in *Platycodon grandiflorum* (Jacq.) A. DC.

**DOI:** 10.3390/plants13202845

**Published:** 2024-10-11

**Authors:** Woo Seok Ahn, Yun Chan Huh, Cheong A Kim, Woo Tae Park, Jang Hoon Kim, Jin-Tae Jeong, Mok Hur, Jeonghoon Lee, Youn-Ho Moon, Sung-Ju Ahn, Tae Il Kim

**Affiliations:** 1 Department of Herbal Crop Research, NIHHS, RDA, Eumseong 27709, Republic of Korea; haploid@korea.kr (W.S.A.); wmelon@korea.kr (Y.C.H.); godcap@korea.kr (C.A.K.); harusarinamu@korea.kr (W.T.P.); jhkim53@korea.kr (J.H.K.); powjjt@korea.kr (J.-T.J.); mok0822@korea.kr (M.H.); artemisia@korea.kr (J.L.); yhmoon@korea.kr (Y.-H.M.); 2 Department of Bioenergy Science and Technology, Chonnam National University, Gwangju 61186, Republic of Korea; asjsuse@jnu.ac.kr

**Keywords:** bell flower, shed-microspore culture, embryogenesis, haploid, regeneration

## Abstract

Anther and microspore cultures are efficient methods for inducing haploids in plants. The microspore culture by chromosome-doubling method can produce double haploid lines, developing pure lines within the first or second generations. This study aimed to induce haploid plants in *Platycodon grandiflorum* using the shed-microspore culture method. *P. grandiflorum* floral buds (n = 1503) were cultured in six types of medium to induce haploids. Anthers were placed on a solid–liquid double-layer medium and cold pre-treated at 9 °C for one week, followed by incubation in the dark at 25 °C. Embryogenesis was observed after approximately 70 days of culture, producing haploid plants through regeneration. Of the 1503 floral buds, embryos developed in 120 buds, resulting in the induction of 402 individuals. Among the media used, Schenk and Hildebrandt (SH) and 1/2SH exhibited high efficiency, with embryogenesis ratios of 12% and 13.4%, respectively. Additionally, the highest embryogenesis ratio (15.3%) was observed in flower buds sized 10 mm or less. Therefore, we established shed-microspore culture conditions to induce haploids in *P. grandiflorum*. Using this method, haploids can be efficiently induced in *P. grandiflorum*, shortening the breeding period by enabling the rapid development of inbred lines.

## 1. Introduction

*Platycodon grandiflorum*, commonly known as bellflower, is a perennial herb belonging to the Campanulaceae family. It has 18 chromosomes (2*n* = 18) and is native to and cultivated in Asia for culinary and medicinal purposes. The flowers bloom from July to August and are purple or white, with five stamens and one pistil. *P. grandiflorum* exhibits protandry, where the stamens mature before the pistil, leading to cross-pollination. Thus, maintaining uniformity in cultivars is challenging, and plant development requires significant time and effort because of its higher genetic variation compared to self-pollinating plants. Therefore, it is necessary to develop techniques such as microspore culture that can shorten the breeding period and cultivate superior and diverse fixed lines [1]. Production of haploids through gametic embryogenesis enables the development of completely homozygous lines from heterozygous parents, enabling the rapid selection of homozygous lines with fixed traits in a shorter period than in traditional breeding, which requires several generations of self-pollination to develop genetically fixed pure lines [2,3]. Since the observation of natural haploids in *Datura stramonium* in 1922, haploid technology has been introduced into plant breeding research and utilized for cultivar development in various crops [4,5,6]. However, inducing haploids involves several factors such as growing donor plants, harvesting floral organs, isolating microspores, culturing and inducing microspores, and regenerating embryos [7].

Several methods can be used to produce haploid plants using microspore culture. Anther culture, first reported in *Datura* in 1964, is the most widely used method of haploid induction [8]. However, anther cultures include somatic tissues that can lead to the induction of somatic calli and diploids, in addition to haploids. These somatic tissues can negatively affect the culture by releasing phenolic compounds, making the selection of haploids more challenging [7]. In 1974, successful microspore culture without anther tissue was achieved in *Nicotiana* [9], following which Lichter [10] initiated research on isolated microspore culture by mechanically separating microspores in *Brassica*. Subsequently, numerous studies have been conducted on isolated microspore cultures in various crops, including pepper [11], rapeseed [12], and asparagus [13]. However, isolated microspore cultures involve a complex process of separating microspores, which can cause considerable contamination.

Shed-microspore culture involves culturing anthers on a solid–liquid double-layer medium in which the osmotic pressure of the liquid medium causes the anther sutures to split, releasing the microspores into the liquid medium for embryo formation. This helps to distinguish between somatic embryos and microspore-derived embryos, even if somatic embryos develop from the anther walls. Shed-microspore culture is a more efficient method for selecting microspore-derived embryos than anther culture, and it has been described in several plants, including Indonesian chili pepper [14], barley [15], and tomato [16]. To date, haploid embryos and plants have been induced in *P. grandiflorum* using anther cultures [17,18,19]; however, there have been no studies on haploid induction through microspore culture in this species.

In the present study, we aimed to produce haploids in *P. grandiflorum* using shed-microspore culture to obtain microspore-derived embryos and plants. We investigated the effects of various media and flower bud sizes on microspore embryogenesis and established the optimal culture conditions for this method. Additionally, we analyzed putative haploid plants obtained through shed-microspore culture for their ploidy levels using flow cytometry.

## 2. Results

### 2.1. Male Gametophyte Development in P. grandiflorum

**Figure 1:**
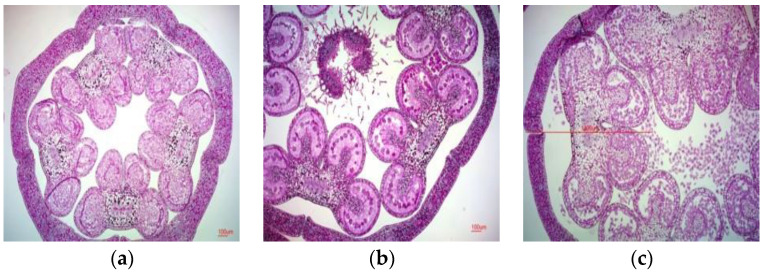
Pollen mother cell development stage of *P. grandiflorum*: (**a**) meiosis (anther size, 2.5 mm); (**b**) tetrad (anther size, 3.1 mm); (**c**) released microspore (anther size, 3.5 mm). Scale bars: 100 μm.

As plant responses to anther or microspore culture can vary, the primary issue was to determine the appropriate stage of pollen mother cell development and to select the correct flower bud size for culture. To establish the criteria for determining the optimal stage to harvest flower buds for microspore culture, the flower bud sizes were measured (Table 1), after removing the sepals and unnecessary tissues. Anthers were collected from buds of different sizes to determine the developmental stage of the pollen mother cells (Figure 1). Flower bud sizes were 4.69 ± 0.53 mm on 17–19 preflowering days, 7.62 ± 1.74 mm on 14–16 preflowering days, 10.62 ± 1.83 mm on 11–13 preflowering days, 13.65 ± 1.79 mm on 8–10 preflowering days, 17.9 ± 2.42 mm on 5–7 preflowering days, and 23.78 ± 3.34 mm on 2–4 preflowering days. Measurement of the anther size during the developmental stages of pollen mother cells in bellflower revealed that the size was 2.5–3.0 mm during the leptotene stage, 3.1–3.4 mm during the tetrad stage, and 3.5 mm during the microspore stage. Floral buds before the tetrad stage were used for shed-microspore culture.

### 2.2. Shed-Microspore Culture for the Induction of Haploid P. grandiflorum

**Figure 2:**
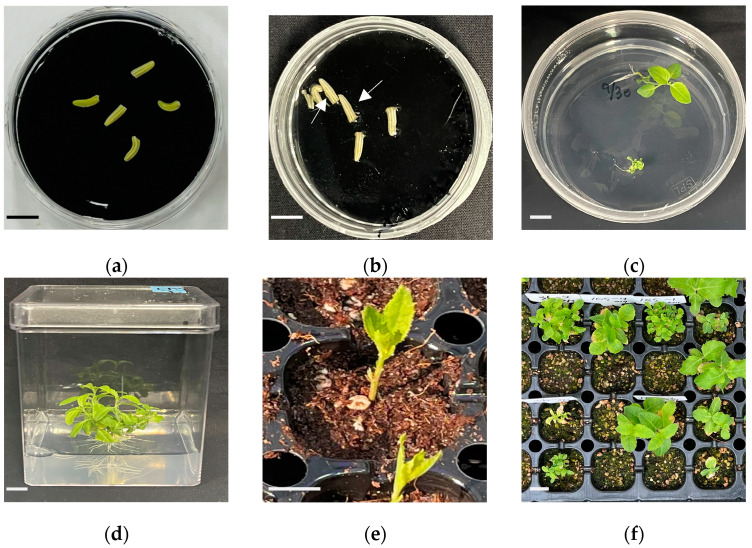
*P. grandiflorum* shed-microspore culture and regeneration process: (**a**) shed-microspore culture in double layer medium; (**b**) embryogenesis after 70 days of shed-microspore culture; (**c**) regeneration from embryos; (**d**) induction of root and whole plant regeneration; (**e**) soil acclimation of regenerated plants; and (**f**) haploid predicted plants. Arrows indicate embryos induced through shed-microspore culture. Scale bars: 1 cm.

To induce haploids, shed-microspore culture was performed using five breeding lines that are not yet commercialized, developed by the Department of Herbal Crop Research at the National Institute of Horticultural and Herbal Science (NIHHS, RDA, Eumseong, Republic of Korea). Shed-microspore culture was performed using a double-layer medium consisting of a solid bottom layer and a liquid top layer (Figure 2a). The embryos were observed after approximately 70 days of culture (Figure 2b), and the developed embryos were transferred to 1/2 Murashige and Skoog (MS) medium (Figure 2c). Root induction was achieved after approximately 4 weeks of culture on 1/2MS medium (Figure 2d). To prevent excessive water loss during soil acclimatization, leaves and parts of the lateral branches were removed, leaving only the true leaves (Figure 2e). Haploid-presumed plants were obtained after approximately 4 weeks of soil acclimatization (Figure 2f).

### 2.3. Effect of Different Media on Embryogenesis

**Figure 3:**
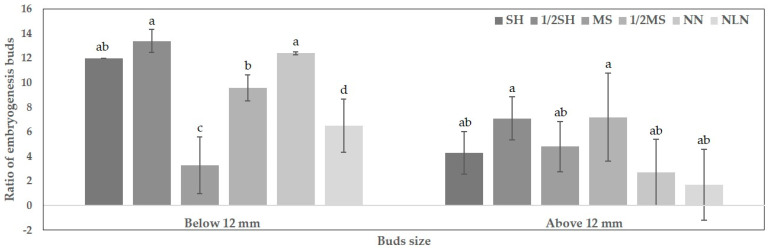
Ratio of embryogenesis buds in buds sized 12 mm or less and 12 mm or more. Means sharing the same letter in a column were not significantly different in Duncan’s multiple comparison range test (*p* < 0.05). Error bars represent standard deviation.

To investigate the effect of different medium types on embryo development with line 20PG004, a breeding line developed by the NIHHS, experiments were performed using six types of medium: SH, 1/2SH, MS, 1/2MS, NN, and NLN (Table 2). Flower buds used in the cultures were divided into two size categories: 12 mm or less, and 12 mm or more (Table 2). Embryogenesis began after 70 days of culture. The ratio of embryogenic buds to the total number of cultured buds, as well as the number of embryogenic buds, was recorded. Embryogenesis was observed in all six types of medium; however, there were differences in the embryogenesis rates. For flower buds 12 mm or less, the highest embryogenesis ratio was observed in the 1/2SH medium at 13.4%, followed by the SH medium at 12% (Figure 3). Similar to the ratio of embryogenic buds, the number of embryos was also high, with 64 embryos observed in the SH medium and 74 in the 1/2SH medium (Table 2). However, for flower buds sized 12 mm or more, a relatively lower ratio of embryogenic buds was observed compared to that of smaller buds (Table 2). In summary, these results indicated that the SH and 1/2SH media using SH salt were the most effective for embryogenesis in bellflower shed-microspore cultures. Furthermore, differences in embryogenesis were observed depending on the flower bud size.

### 2.4. Effect of Flower Bud Size on Embryogenesis

**Figure 4:**
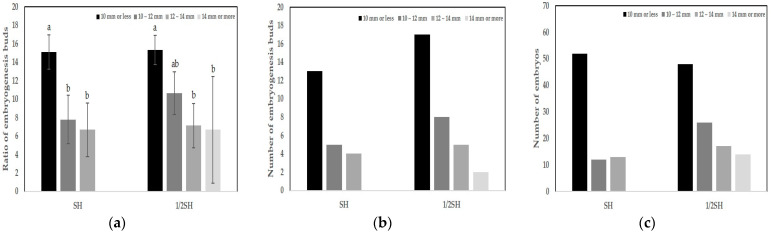
Embryogenesis and bud size in *P. grandiflorum*; bud sizes were 10 mm or less, 10–12 mm, 12–14 mm, and 14 mm or more. (**a**) Ratios of embryogenic buds were determined by counting the total numbers of buds with embryogenesis relative to the total number of buds cultured. (**b**) Number of embryogenic buds from total cultured buds. (**c**) Number of embryos from total cultured buds. Means sharing the same letter in a column were not significantly different in Duncan’s multiple comparison range test (*p* < 0.05). Error bars represent standard deviation.

The effect of flower bud size on embryogenesis in shed-microspore culture was also investigated. Flower buds, cultured in the SH and 1/2SH media, exhibited high embryogenesis efficiency. These buds were divided into four size categories: 10 mm or less, 10–12 mm, 12–14 mm, and 14 mm or more. The number of embryogenic buds was the highest when the flower bud size was 10 mm or less, with 13 buds in the SH medium and 17 buds in the 1/2SH medium (Table 3, Figure 4b), and a ratio of embryogenic buds of 15.1% in the SH medium and 15.3% in the 1/2SH medium (Figure 4a). The number of embryos obtained from each bud was the highest when the flower bud size was 10 mm or less, with 52 embryos in the SH medium and 48 embryos in the 1/2SH medium (Table 3, Figure 4c). However, for flower buds of 14 mm or more, an embryogenesis ratio of only 6.9% was observed in the 1/2SH medium, whereas no embryogenesis was observed in the SH medium (Figure 4a). In addition, the number of embryogenic buds and embryos according to lineage were investigated in the SH medium. All three lineages exhibited a high number of embryogenesis buds in flower buds sized 10 mm or less and 10–12 mm. In particular, in the case of 20PG43-3, similar embryogenesis results were observed even in flower buds sized 12–14 mm compared to those sized 10–12 mm (Table 4). In summary, in bellflower shed-microspore culture, there may be differences in embryogenesis rates depending on the lineage, but buds sized 10 mm or less were the most effective for embryogenesis. In addition, as bud size increased, embryogenesis either decreased or did not occur.

### 2.5. Ploidy Analysis and Characterization of Regenerated Plants

**Figure 5:**
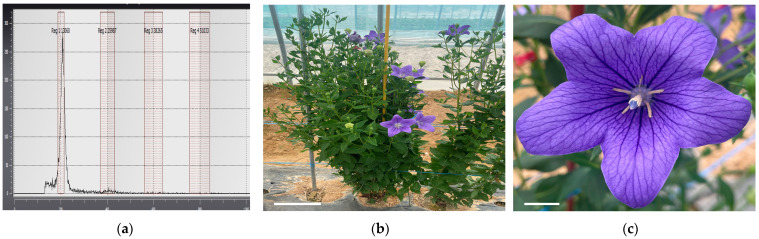

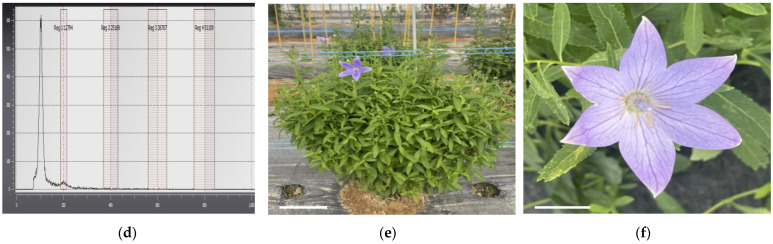
*P. grandiflorum* diploid (control), haploid ploidy analysis and plants characteristics: (**a**) flow cytometry histogram of diploid (control); (**b**) diploid plants; (**c**) flower with normally produced pollen; (**d**) flow cytometry histogram of haploid; (**e**) haploid plants; and (**f**) flowers of haploid without pollen. Scale bars represent 10 cm (**b**,**d**) and 1 cm (**c**,**f**).

**Figure 6:**
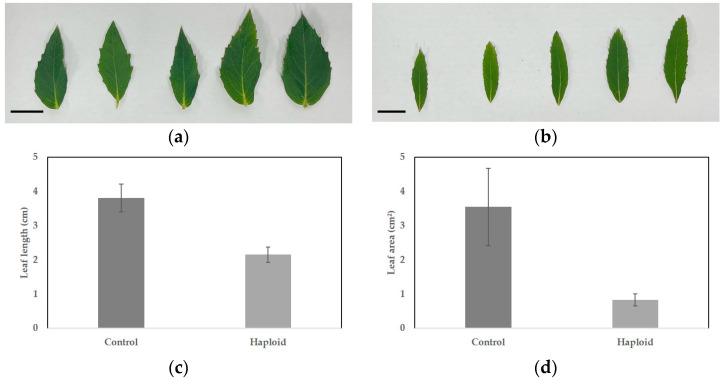
Leaf length (cm) and area (cm^2^) of diploid and haploid *P. grandiflorum*: (**a**) diploid leaf (control); (**b**) haploid leaf; (**c**) leaf length in diploid and haploid; and (**d**) leaf area in diploid and haploid. Scale bars represent 1 cm. Different letters are significantly different at 95% level by Duncan’s test. Error bars represent standard deviation.

Plants obtained through shed-microspore culture were subjected to ploidy analysis using flow cytometry (Figure 5a,d). Through shed-microspore culture, 120 putative haploid *P. grandiflorum* plants were induced. Flow cytometry analysis confirmed that 65 of these plants were haploids, accounting for 54.2% of the total, whereas the remaining plants were identified as chimeras or diploids. After soil acclimatization, the haploid plants exhibited significant differences compared to the diploid plants (Figure 6a,b). The leaf length of the diploid plants was 3.81 cm, whereas the haploid plants had shorter leaves of 2.15 cm (Table 5, Figure 6c). The leaf area of diploid plants was measured at 3.55 cm^2^, whereas haploid plants had a smaller leaf area of 0.83 cm^2^, indicating that haploid plants were smaller compared to diploid plants (Table 5, Figure 6d). Overall, the haploid plants exhibited smaller leaves and more lateral branches than the diploid control group (Figure 5b,e). In addition, diploid plants produced normal flowers with pollen, whereas haploid plants either did not form pollen or did not bloom (Figure 5c,f).

### 2.6. Comparison of Stomatal Size and Frequency between Diploid Control and Haploid P. grandiflorum

**Figure 7:**
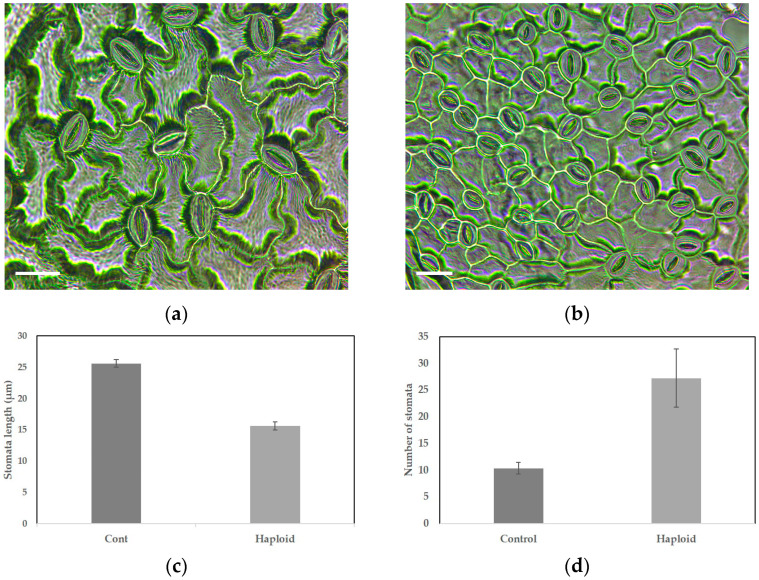
Stomatal characteristics of diploid and haploid *P. grandiflorum***:** (**a**) diploid stomata (control); (**b**) haploid stomata; (**c**) stomata length in diploid and haploid; (**d**) number of stomata in diploid and haploid. Scale bars represent 20 μm. Different letters are significantly different at 95% level by Duncan’s test. Error bars represent standard deviation.

The stomata size of diploid plants averaged 25.62 ± 0.22 μm, whereas haploid plants exhibited a smaller stomata size, averaging 15.63 ± 0.24 μm compared to the diploid control group (Table 6, Figure 7). However, the number of stomata per unit area was approximately 2.6 times higher in haploid plants, with 27.25 stomata, compared to 10.38 stomata in the diploid control plants (Table 6, Figure 7).

## 3. Discussion

In the present study, we developed an efficient shed-microspore culture method for inducing haploids in bellflowers.

The developmental stage of microspores is crucial for embryogenesis and can vary among species [7]. In addition, flower bud size can significantly affect embryogenesis [20]. According to Supena [14], selecting flower buds at the late uninucleated phase is a key factor for successful anther culture. Embryogenesis was higher during the transition from the late uninucleate to early binucleate stages [21]. The size and shape of flower buds can be used as indirect indicators to determine the developmental stage of microspores. Therefore, we used flower buds during embryogenesis (Table 1, Figure 1). There is a close relationship between flower bud size and the developmental stage of immature pollen. Therefore, haploid plantlets are more likely to be induced in flower buds of an appropriate size, corresponding to the stage between the start of meiosis and the pre-microspore stage.

Culture medium plays an important role in inducing embryogenesis in microspores and anther cultures. The most commonly used basal media are N6 [22], B5 [23], and MS [24], which have varying efficiencies in inducing embryogenesis depending on the species. For example, *Brassica* species efficiently undergo embryogenesis in NLN or modified NLN media [25], *Solanaceae* generally use half-strength MS medium, and *Poaceae* crops utilize N6 medium [26]. The carbon source added to the medium is essential for embryogenesis because of its osmotic and nutritional effects [27]. In pepper microspore culture, the frequency of embryogenesis was highest when sucrose was used as the carbon source, compared to maltose [11]. However, in barley, among the carbon sources, sucrose, glucose, and fructose did not induce embryogenesis and led to cell death, whereas, only maltose induced embryo formation [28]. Lactose and galactose are typically used in clementine anther cultures [29], but sucrose was found to be the optimal carbon source for two clementine and two mandarin varieties [30]. As the medium is a crucial factor for inducing embryogenesis, this study investigated the embryogenesis efficiency of *P. grandiflorum* by culturing in six different media, including SH. As shown in Table 2, there were differences in embryogenesis efficiency across the media; however, embryogenesis occurred in all six media, with the highest efficiency of 13.4% observed in the 1/2SH medium. This indicates that the type and composition of the medium affect microspore embryogenesis in *P. grandiflorum*.

The results obtained in this study clearly show that shed-microspore culture can be an attractive technique for haploid production in bellflower. We plan to establish various conditions to enhance haploid induction and conduct research on the production of doubled haploid lines using these methods to develop new cultivars.

## 4. Materials and Methods

### 4.1. Plant Materials

In this study, five lines of *P. grandiflorum* (Jacq.) A. DC. were used, which were developed by the Department of Herbal Crop Research at the National Institute of Horticultural and Herbal Science (NIHHS, RDA, Eumseong, Republic of Korea). The seeds of these *P. grandiflorum* lines were stored in the seed storage facility at the Department of Herbal Crop Research, NIHHS. The seeds were sown in 128-cell seedling trays filled with Baroker Horticultural Soil (Seoul Bioscience, Eumseong, Republic of Korea). When the third or fourth true leaf appeared around the end of April, 30 plants per progeny were transplanted to a greenhouse. The greenhouse comprised four 120 cm wide ridges, each covered with black plastic mulch. Flower buds began to form in mid-June and continued flowering until mid-September. Suitable buds were then collected and cultured.

### 4.2. Cold Pretreatment and Sterilization

Bellflower buds sized 9–15 mm, which had not yet bloomed, were harvested in the morning (at 9 a.m.) and pre-treated at 4 °C for 24 h in a low-temperature chamber. After removing the sepals and unnecessary tissues, the buds were placed in 50 mL tubes, sterilized with 70% ethanol for 30 s, rinsed once with sterilized distilled water, and shaken in a 2% NaOCl solution for 15 min for sterilization. The buds were then rinsed five times with sterilized distilled water. Moisture on the surface-sterilized buds was removed using a sterilized filter paper before the experiments.

### 4.3. Shed-Microspore Culture

Six types of double-layer medium were used for the shed-microspore culture, each comprising a solid bottom layer and a liquid top layer. The solid bottom-layer media used were SH and 1/2SH [31], MS and 1/2MS [25], NN [32], and NLN [10], each containing 2% sucrose and 1% activated charcoal, and solidified with 0.4% Gelrite. The liquid top layer had the same composition as the solid bottom layer, including only 2% sucrose. Both layers were adjusted to pH 5.8 and sterilized by autoclaving. The solid bottom layer was dispensed into 60 × 15 mm Petri dishes to form the cultures. The anthers extracted from the bellflower buds were spread on the solid bottom layer, and 1.5 mL of the liquid top layer medium was added. The Petri dishes were cold-treated at 9 °C for 7 days before being transferred to 26 ± 2 °C for embryogenesis. After 2 weeks of culture, an additional 1.5 mL of the liquid top-layer medium was added.

### 4.4. Plant Regeneration and Soil Acclimatization

Embryos induced through shed-microspore culture were transferred to MS medium containing 2% sucrose and 0.4% Gelrite, then incubated in a growth chamber at 26 ± 2 °C for plant regeneration. Shoots derived from the embryos were transferred to square culture dishes (75 mm × 75 mm × 100 mm) to facilitate root development and continued growth. When the shoots reached a length of approximately 5–6 cm, soil sterilization was performed by gently rinsing the roots under running water to remove as much adhering medium as possible to prevent root detachment. The plantlets were transplanted after filling a 105-cell seedling plug tray with moistened soil. The tray lids were opened from 9 a.m. to 12 p.m. to allow ventilation, and moisture was replenished through the bottom of the tray using a capillary mat system.

### 4.5. Ploidy Analysis

Young leaves from each plant were used for ploidy analysis. Leaf tissue was placed in a Petri dish, and 0.25 mL of Cystain™ UV Precise P Nuclei Extraction Buffer (Sysmex Partec GmbH, Arndtstraße 11 a-b, 02826 Goerlitz, Germany) was added. The tissue was cut with a razor blade; the extraction buffer containing the released nuclei was filtered using a 30 µm CellTrics™ nylon filter (Sysmex Partec GmbH, Arndtstraße 11 a-b, 02826 Goerlitz, Germany) attached to a 3.5 mL sample tube. Filtered extracts were stained by adding 1 mL of Cystain™ UV Precise P Staining Buffer, followed by flow cytometry analysis. DNA peaks were measured against standard diploid bellflowers, and ploidy levels (haploid or diploid) were determined by comparing the peaks with the standard peak. Ploidy analysis was performed using a CyFlow Ploidy Analyzer (Sysmex Partec GmbH, Arndtstraße 11 a-b, 02826 Goerlitz, Germany) equipped with UV (365 nm) and green (532 nm) lasers. Nuclei peak positions were determined and analyzed using the CyView software(version number CyView 1.6)—a Flow Cytometry Standard (FCS) file analysis software based on the Windows™ platform.

### 4.6. Stomatal Observation

To compare the size and density of stomata between the diploid control and haploid plants, the nail polish impression method was used [33]. A transparent nail polish was applied to the back of the leaves and allowed to dry for approximately 10 min. Next, the nail polish was removed from the leaves using transparent tape, and the tape was attached to a glass slide to obtain the stomatal samples. The stomata were observed under an optical microscope at a magnification of ×400. Five stomata per plant were measured in each treatment group, with 10 plants per treatment group.

### 4.7. Statistical Analysis

Statistical analyses were performed using the Statistical Analysis System (SAS) Enterprise Guide 7.1 (SAS, 2009; SAS Institute Inc., Cray, NC, USA) to calculate the mean, standard deviation, and analysis of variance. After conducting a significance test on the analysis results, statistical significance was verified using Duncan’s Multiple Range Test at a 5% significance level (*p* < 0.05).

## 5. Conclusions

In this study, we successfully established an efficient shed-microspore culture technique for haploid induction in *P. grandiflorum* for the first time. The shed-microspore culture is relatively simple, has a high haploid induction rate, and enables rapid development of homozygous lines, which can significantly reduce the time required for breeding.

## Figures and Tables

**Table 1:** Flower bud size and anther size by flowering stage of *Platycodon grandiflorum*.

Flowering Stage (Days before Flowering)	Bud Size (mm)	
	Whole Bud	Anther
17–19	4.69 ± 0.53	2.6 ± 0.26
14–16	7.62 ± 1.74	3.81 ± 0.6
11–13	10.62 ± 1.83	5.46 ± 0.59
8–10	13.65 ± 1.79	6.88 ± 0.58
5–7	17.9 ± 2.42	8.03 ± 0.46
2–4	23.78 ± 3.34	8.42 ± 0.62

Values are presented as mean ± SE (standard error) of three independent experiments with three replicates each.

**Table 2:** Number of embryogenesis buds and embryos by medium in shed-microspore culture.

Medium	12 mm or Less			12 mm or More		
	Number of Buds in Culture	Number of Embryogenesis Buds	Number of Embryos	Number of Buds in Culture	Number of Embryogenesis Buds	Number of Embryos
SH	150	18	64	94	4	13
1/2SH	186	25	74	99	7	31
MS	149	5	66	84	4	6
1/2MS	178	17	31	83	6	11
NN	170	21	75	112	3	7
NLN	138	9	23	60	1	1

Values in the table represent the number of embryogenic floral buds and number of embryogenic individuals relative to the total number of cultured floral buds.

**Table 3:** Number of embryogenic buds and number of embryos based on floral bud size.

Bud Size (mm)	SH			1/2SH		
	Number of Buds in Culture	Number of Embryogenesis Buds	Number of Embryos	Number of Buds in Culture	Number of Embryogenesis Buds	Number of Embryos
10 or less	86	13	52	111	17	48
10–12	64	5	12	75	8	26
12–14	60	4	13	70	5	17
14 or more	34	0	0	29	2	14

Values in the table represent the number of embryogenic floral buds and number of embryogenic individuals relative to the total number of cultured floral buds.

**Table 4:** Embryogenesis buds and embryos by medium in shed-microspore culture.

Medium	Line	Bud Size (mm)	Number of Buds in Culture	Embryogenesis Results	
				Number of Buds	Number of Embryos
SH	20PG43-3	10 or less	119	15	40
		10–12	114	26	141
		12–14	50	25	130
		14 or more	10	0	0
	20PG98-1	10 or less	19	8	75
		10–12	14	7	42
		12–14	14	1	1
		14 or more	4	0	0
	20PG006	10 or less	20	3	13
		10–12	15	2	4
		12–14	18	3	9
		14 or more	13	0	0

Values are the number of embryogenic buds and the number of embryos relative to the total number of cultured floral buds.

**Table 5:** Leaf length and area of diploid and haploid *P. grandiflorum*.

Ploidy Level	Leaf Length (cm)	Leaf Area (cm^2^)
Diploid (control)	3.81 ± 0.13 ^a^	3.55 ± 0.36 ^a^
Haploid	2.15 ± 0.07 ^b^	0.83 ± 0.06 ^b^

Values are provided as mean ± S. D. and those followed by different letters are significantly different at 95% level by Duncan’s test.

**Table 6:** Stomata length and number of stomata in diploid and haploid *P. grandiflorum*.

Ploidy Level	Stomata Length (μm)	Number of Stomata
Diploid (control)	25.62 ± 0.22 ^a^	10.38 ± 0.42 ^a^
Haploid	15.63 ± 0.24 ^b^	27.25 ± 2.05 ^b^

Data are provided as mean ± S. D. and those followed by different letters are significantly different at 95% level by Duncan’s test.

## Data Availability

The data that support the findings of this study are available from the corresponding author upon reasonable request.
